# Invasive Klebsiella pneumoniae Syndrome: A Case Report From Malaysia

**DOI:** 10.7759/cureus.71814

**Published:** 2024-10-18

**Authors:** Xian Pei Cheong, Li Min Lim, Chee Yik Chang

**Affiliations:** 1 Internal Medicine, Hospital Sultanah Aminah, Johor Bahru, MYS; 2 Infectious Diseases, Hospital Sultanah Aminah, Johor Bahru, MYS

**Keywords:** cavitating lung lesions, hypervirulent k. pneumoniae (hvkp), invasive klebsiella syndrome, klebsiella pneumoniae (kp), metastatic klebsiella syndrome

## Abstract

Invasive Klebsiella syndrome is a potentially life-threatening condition primarily caused by hypervirulent strains of *Klebsiella pneumoniae*. It is characterized by severe infections that metastasize to various organs, including the liver, lungs, eyes, and brain. We present a case of invasive *K. pneumoniae* syndrome in Malaysia, highlighting the aggressiveness of the disease. The case involves a 44-year-old woman with diabetes mellitus who developed cavitary pneumonia, pulmonary embolism, and pleural effusion, requiring prolonged antibiotic treatment and drainage. This case highlights the need for early diagnosis and extended antibiotic therapy to improve patient outcomes.

## Introduction

Invasive Klebsiellasyndrome (IKS) is a clinical condition characterized by severe, life-threatening infections predominantly caused by hypervirulent strains of *Klebsiella pneumoniae*. This condition is known for its ability to metastasize to different organs, including the liver, lungs, eyes, and brain [1].

IKS is an emerging infectious disease, particularly in the Asia region. An epidemiological study conducted in South Korea revealed that out of 371 cases of liver abscess, 290 (78.2%) were attributed to *K. pneumoniae*, with distant metastatic infections observed in 8.7% of cases [2]. In Taiwan, metastatic infections were detected in 17 patients (15.5%), with meningitis in 11 patients (64.7%) and endophthalmitis in 4 patients (23.5%). The overall mortality rate was 10% [3]. A local study found that *K. pneumoniae *was the most common pathogen causing endogenous endophthalmitis (32.5%) [4].

Hypervirulent *K. pneumoniae *(hvKp) can cause a variety of serious infections in both healthy individuals and hospitalized patients. In contrast, classical *K. pneumoniae *(cKp) primarily causes healthcare-associated infections. Accurate identification of hvKp is critical for clinical practice to improve patient outcomes. Hypervirulent *K. pneumoniae *can be differentiated from classical strains by the string test, which assesses hypermucoviscosity, as well as via polymerase chain reaction for the detection of virulence-associated genes [5].

This case report aims to provide an overview of IKS by presenting clinical, radiological, and microbiological data of a patient with IKS. We hope that by analyzing this case, we can highlight the common clinical features, diagnostic challenges, and treatment outcomes of IKS.

## Case presentation

A 44-year-old woman with underlying diabetes mellitus presented with a 10-day history of fever, left flank pain, dysuria, cough, and pleuritic chest pain. Her vital signs on arrival to the emergency department were: blood pressure of 144/94 mmHg, heart rate of 101 beats per minute, temperature of 39.5 °C, and oxygen saturation of 98% on room air. A chest radiograph revealed bilateral lower zone consolidation. Blood tests showed an elevated C-reactive protein (CRP) level of 96.7 mg/L (normal range: <5 mg/L) and a normal white cell count of 6.7 × 10⁹/L (normal range: 4-10 × 10⁹/L). She was treated for a chest infection and urinary tract infection, and intravenous amoxicillin-clavulanic acid 1.2 g every 8 hours and oral azithromycin 500 mg daily were initiated, with blood cultures taken.

After 48 hours of incubation, blood cultures revealed the presence of *K. pneumoniae*, which was susceptible to ampicillin, ampicillin-sulbactam, amoxicillin-clavulanic acid, gentamicin, carbapenems, ceftriaxone, sulfamethoxazole-trimethoprim, and ciprofloxacin. The string test was negative. Further molecular analysis of the isolate confirmed hypervirulent *K. pneumoniae* with the *iroD*, *rmpA*, and *peg* genes detected, but the *magA* gene was not detected.

**Figure 1:**
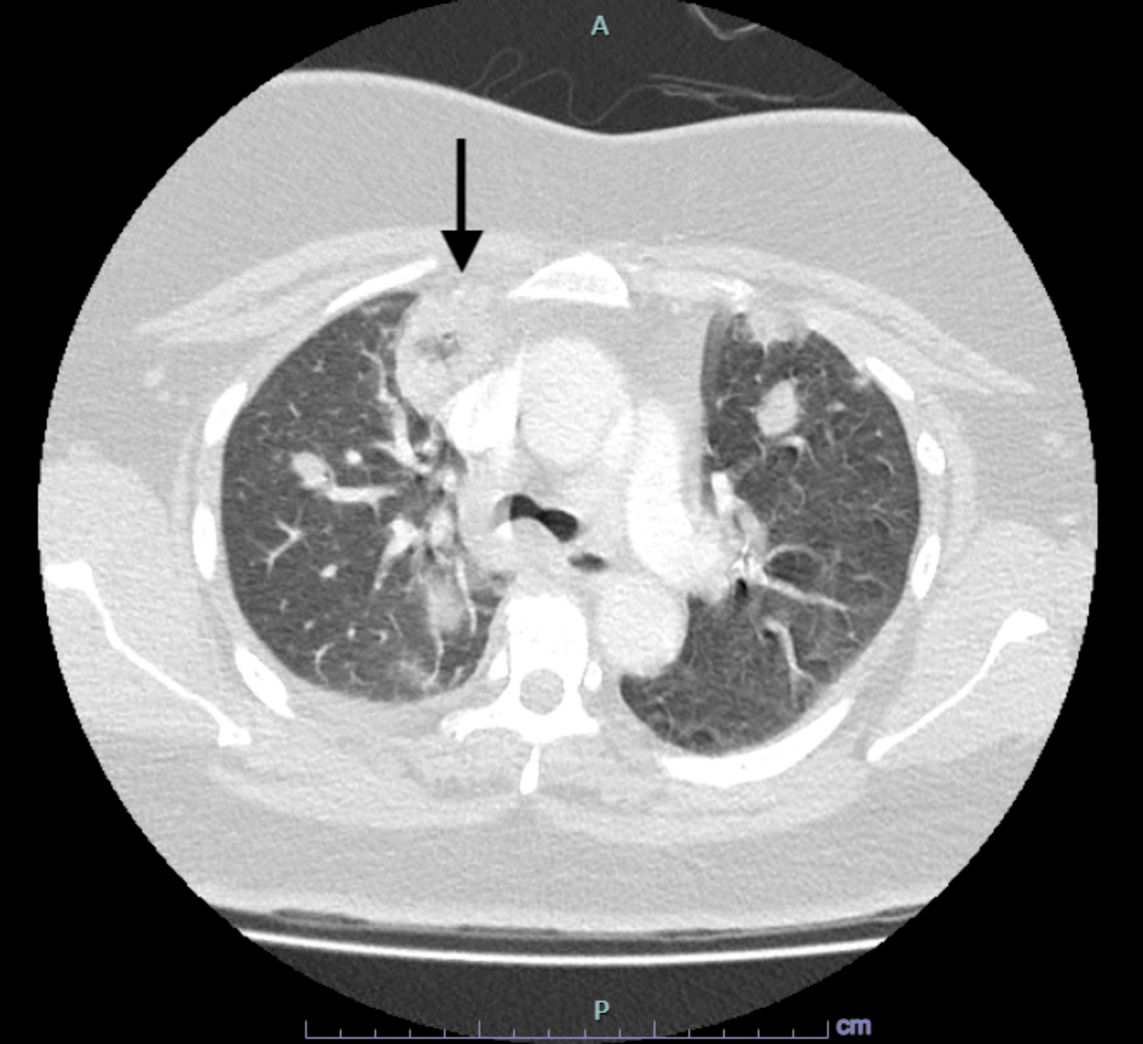
Initial CT scan of thorax showing bilateral cavitary pneumonia with predominant nodule at the right upper lobe, measuring 3.5 x 2.7 x 2.3 cm. A: anterior, P: posterior.

On day 5 of admission, the patient deteriorated and developed hypoxic respiratory failure, necessitating supplemental oxygen via Venturi mask. Antibiotic therapy was escalated to intravenous piperacillin-tazobactam 4.5 g every six hours to empirically treat nosocomial pneumonia. Due to her sudden deterioration and increasing tachycardia, a CT pulmonary angiogram was performed, revealing bilateral small subsegmental pulmonary emboli, bilateral cavitary pneumonia, and a predominant cavitating lesion in the right upper lobe measuring 3.5 × 2.7 × 2.3 cm, along with a loculated effusion on the right side (Figure 1). The echocardiogram was normal, with a normal ejection fraction.

An ultrasound-guided pigtail catheter was inserted, and the pleural fluid was exudative in nature. Fluid analysis revealed fluid albumin at 18 g/L, total protein at 54 g/L, lactate dehydrogenase (LDH) at 839 U/L, amylase at 46 U/L, and glucose at 3.8 mmol/L. The pleural fluid to serum LDH ratio was 2, and the pleural fluid to serum protein ratio was 0.8. Gram stain showed a few pus cells but no organisms, with the fluid appearing slightly blood-stained. The cell count was 15 white blood cells, with 67% polymorphs and 33% lymphocytes, along with numerous red blood cells. Pleural fluid culture revealed no growth.

The pigtail catheter was removed after one week as the drain output became minimal. As the patient’s condition improved, antibiotics were de-escalated to intravenous ceftriaxone 1 g twice daily for five more days, followed by intravenous amoxicillin-clavulanic acid 1.2 g every eight hours for an additional nine days. She was later discharged with a plan to receive intravenous ceftriaxone 2 g daily for two weeks through the outpatient antibiotics therapy (OPAT) program. She was also treated with oral anticoagulants for a total of three months.

**Figure 2:**
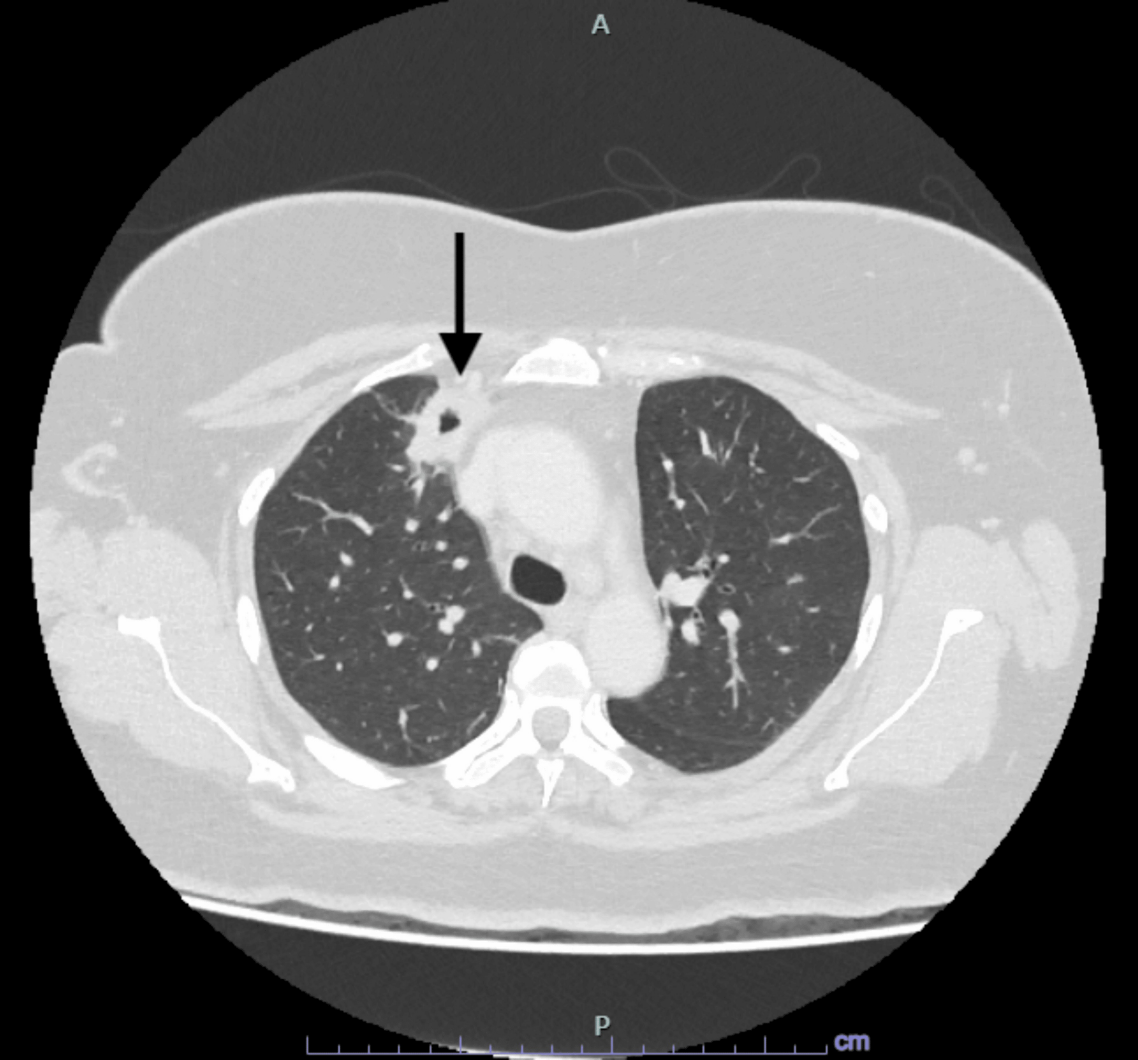
Interval CT Scan of thorax after four weeks of effective antibiotics showing significant improvement with a smaller nodule at the right upper lobe, measuring 2.8 x 1.7 x 3 cm. A: anterior, P: posterior

After two weeks of intravenous ceftriaxone, completing four weeks of total effective antibiotic therapy, a contrast-enhanced CT scan of the thorax, abdomen, and pelvis revealed a thick-walled cavitating lesion in the right upper lobe, measuring 2.8 × 1.7 × 3 cm (Figure 2). Overall, the lung findings indicated improvement, with residual lung infection and a thin, complex right pleural effusion, while the patient’s symptoms improved significantly.

**Figure 3:**
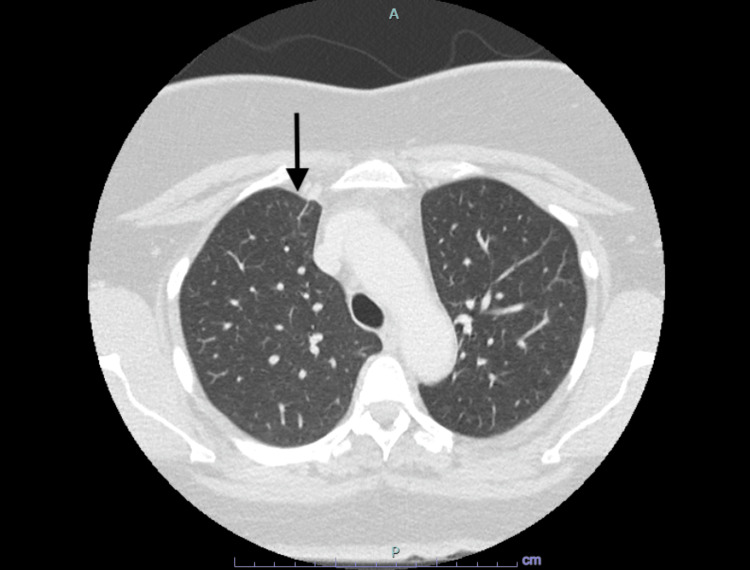
CT scan of thorax after 12 weeks of effective antibiotics showing resolving lung infection with resolved right upper lobe lesion. A: anterior, P: posterior.

She was given oral levofloxacin 750 mg daily, and a repeat CT scan eight weeks later, that is, after completing 12 weeks of total effective antibiotics, revealed that the right upper lobe lesion had resolved, as shown in Figure 3. Since she was clinically well, antibiotics were discontinued after completing the 12-week course of effective therapy.

## Discussion

IKS, characterized by severe infections such as liver abscesses, pneumonia, and urinary tract infections, is increasingly recognized as a significant clinical challenge, especially in patients with underlying comorbidities like diabetes mellitus. Studies have found that diabetes mellitus is present in 29.3-44.3% of *K. pneumoniae *liver abscess cases [6,7]. Elevated blood sugar levels weaken the immune system, making individuals more susceptible to hvKp infections by impairing neutrophil phagocytosis of K1/K2 *K. pneumoniae *strains [8].

*K. pneumoniae* can cause metastatic infections. Approximately one-third of metastatic infections are detected at the time of admission, with most being diagnosed within three days of the initial presentation [3]. The primary metastatic manifestations include meningitis, endophthalmitis, septic pulmonary emboli, and empyema [9]. A prostate abscess is a rare complication of *K. pneumoniae *infections [10]. However, in Taiwan, *K. pneumoniae *is the most common pathogen associated with prostate abscesses, particularly in diabetic patients [11]. These findings underscore the importance of appropriate and timely imaging for the early detection of metastasis.

Hypervirulent *K. pneumoniae *lacks a precise definition, but strains with virulent capsular types or those carrying the *rmpA/rmpA2 *genes are classified as hypervirulent. These strains, especially those with K1 or K2 capsular types, are generally susceptible to common antibiotics and rarely exhibit resistance, except for their inherent resistance to ampicillin [12]. The string test, which is simple to perform in a laboratory setting, aids in the early detection of hvKp infections. When bacterial colonies on an agar plate are stretched with a bacteriology inoculation loop or needle, a viscous string longer than 5 mm forms [13]. Various virulence factors have been linked to hvKp strains, including capsular serotypes K1 and K2, as well as the mucoviscosity-associated gene A (*magA*) and the regulator of mucoid phenotype A (*rmpA*) genes [14].

This case demonstrates the aggressiveness of invasive Klebsiella infections, as well as the importance of early detection and management. The presence of an underlying condition, such as diabetes, likely contributed to the severity and complexity of the infections. This case also highlights the importance of a multidisciplinary approach, involving infectious disease specialists, microbiologists, and radiologists, to improve patient outcomes.

In recent years, the rise of MDR and hvKp strains has posed a significant health threat, limiting treatment options and leading to higher morbidity and mortality [15,16]. The optimal treatment for MDR *K. pneumoniae *infections remains unclear. Combination therapies, including high-dose meropenem, colistin, fosfomycin, tigecycline, and aminoglycosides, have been used but with suboptimal results [17].

The management of invasive Klebsiella infections requires a high index of suspicion, prompt initiation of appropriate antimicrobial therapy, and thorough investigation to identify and drain abscesses or other foci of infection. Imaging modalities, such as CT scans and ultrasound-guided interventions, play a crucial role in diagnosing and managing these patients. Additionally, the prolonged duration of both intravenous and oral antibiotic therapy underscores the need for sustained treatment to prevent relapse and ensure complete resolution of the infection.

## Conclusions

This case demonstrates the complexity of IKS and the critical need for comprehensive and timely management strategies to improve patient outcomes. The clinical presentation of IKS can vary, ranging from pneumonia to intra-abdominal infections. Therefore, the possibility of IKS should be considered, particularly in patients with poorly controlled diabetes mellitus presenting with sepsis. Further research is warranted to better understand the pathogenesis of this syndrome and to develop more effective therapeutic approaches.
